# Cognitive Load and Working Memory in Multimedia Video Podcasts: Effects of Elaborative and Seductive Details

**DOI:** 10.3390/jintelligence14050074

**Published:** 2026-05-01

**Authors:** Robert O. Davis, Yong-Jik Lee, Joseph Vincent, Ji Hae Lee

**Affiliations:** 1 Department of Education, Chonnam National University, Gwangju 61186, Republic of Korea; rdavis@jnu.ac.kr (R.O.D.); leeji@jnu.ac.kr (J.H.L.); 2 School of Sarim-Honors, Changwon National University, Changwon 51140, Republic of Korea; 3 English for International Conferences and Communication, Hankuk University of Foreign Studies, Seoul 02450, Republic of Korea; vincent@hufs.ac.kr

**Keywords:** video podcasts, working memory, cognitive load, situational interest, multimedia learning, seductive details

## Abstract

Educational video podcasts are increasingly used in higher education, yet the cognitive mechanisms underlying their effectiveness remain underexplored. This experimental study examined how working memory capacity and additive contextual features influence situational interest, cognitive load, and learning from educational video podcasts. Sixty-two university students viewed video podcasts on lightning with standard narration with visuals (control), elaborative details, or seductive details. Working memory did not significantly predict learning outcomes or moderate the effects of additive features, and no differences were observed across conditions for cognitive load or situational interest. In free recall, the control condition outperformed the seductive details condition (*p* = .004, g = 1.03), though this difference was not significant when controlling for working memory. Transfer did not differ across conditions. Follow-up interviews revealed that students across conditions emphasized visuals as critical for comprehension, while those in additive conditions reported feeling overwhelmed despite similar cognitive load ratings. These findings suggest that well-designed multimedia environments may stabilize learning outcomes across differences in working memory capacity, extending cognitive theory of multimedia learning. Designers of educational video podcasts should prioritize narration aligned with supportive visuals over additive contextual features.

## 1. Introduction

Audio-based learning technologies have advanced substantially over the past two decades, moving from simple lecture recordings toward broader instructional uses. In the early 2000s, podcasts emerged as a promising educational medium, driven by portable media players (e.g., iPods), RSS-based subscription technologies, and increasing mobile device ubiquity (Edirisingha et al., 2007; Hew, 2009). Early educational podcasts were primarily audio-only formats enabling students to access lectures or Supplementary Materials offline on personal computers or MP3 players (McGarr, 2009). Audio podcasts helped reduce isolation among distance learners by creating direct, conversational communication with instructors (Nie, 2010). Additionally, students could apply varied learning strategies, including pausing, rewinding, and replaying content to review difficult material, catch up on missed classes, and prepare for examinations through repeated listening. Nevertheless, early studies viewed podcasts as substitutional resources delivering existing lectures rather than intentionally designed instructional techniques with clear pedagogical structures. Hew (2009) highlighted that many studies provided incomplete design descriptions (e.g., omitting duration or podcast types) and focused predominantly on lectures or Supplementary Materials. Similarly, McGarr (2009) observed that higher education podcasting was typically adopted to increase flexibility and accessibility but more often augmented existing lecture-based practice than transformed pedagogy. As podcasting became more widespread, the inability of audio-only formats to convey visual information emerged as a key limitation, contributing to growing interest in multimodal delivery forms such as video podcasts (Hew, 2009; McGarr, 2009; Kay, 2012).

Video podcasts were consistent with established multimedia learning theories and addressed limitations inherent in audio-only podcasts. The cognitive theory of multimedia learning (CTML; Mayer, 2005, 2014) proposes that individuals learn more effectively when information is presented in words and pictures together than words alone, as learning involves processing through separate auditory and visual channels with limited capacity, and meaningful learning depends on actively selecting, organizing, and integrating information (Mayer, 2009). Well-designed video-based instruction can leverage dual channels by coordinating spoken narration with relevant visuals, improving comprehension and transfer while reducing excessive cognitive processing (Mayer, 2014). CTML provides design principles for audio and video presentations, including video podcasts. For example, the modality principle suggests that presenting verbal information as spoken narration rather than on-screen text distributes processing demands across channels, preventing visual channel overload (Mayer, 2005). Video podcasts are particularly well-suited for integrating verbal and visual information into coherent mental representations. Although learners report positive perceptions and some studies indicate performance benefits (Brame, 2017; Anis et al., 2025), empirical evidence remains limited examining how cognitive factors, such as working memory capacity, influence learning with video podcasts.

Research on working memory and cognitive load provides theoretical grounding for understanding these cognitive processes. Despite the promise of dual-channel presentation, the effectiveness of podcasts and video podcasts depends on learners’ working memory capacity and cognitive load management during instruction. A. D. Baddeley and Hitch’s (1974) multicomponent model describes working memory as comprising a phonological loop for processing auditory and verbal information, a visuospatial sketchpad for visual information, and a central executive coordinating these subsystems. The phonological loop is particularly relevant to podcast-based learning, as it temporarily stores and rehearses spoken information through articulatory control processes (A. Baddeley, 2000). However, working memory has limited capacity, typically holding about seven items for fewer than 30 s (Miller, 1956). Cognitive Load Theory (CLT) emphasizes that when instructional demands exceed this capacity, learning is hindered because extraneous load interferes with schema construction and transfer to long-term memory (Sweller, 2011). Effective instruction should reduce extraneous cognitive load and support working memory allocation to learning (Sweller et al., 2019). These considerations are relevant when designing video podcasts by structuring content into manageable segments and enabling learners to pause or replay material. Kay (2012) suggests that well-designed, structured video podcasts support learning by presenting information clearly and in an organized manner.

This exploratory study examines how individual differences in working memory capacity relate to video podcasts that include different types of additive contextual features, such as elaborative and seductive details. Specifically, it investigates how these additive features influence cognitive load, situational interest, and learning outcomes with and without working memory as a covariate. Although video podcasts are widely used in higher education, additive contextual features and core cognitive constructs, such as working memory, cognitive load, and situational interest, have rarely been examined together in multimedia environments. Accordingly, this study aims to clarify whether observed effects in video podcast learning reflect individual differences in cognitive capacity, instructional design features, or a combination of elements. A subset of participants completed follow-up interviews to explore their subjective experiences.

## 2. Literature Review

### 2.1. Working Memory and Cognitive Load

In multimedia learning environments, learners must coordinate verbal and visual inputs to build mental models (Mayer, 2009). Consistent with limited working memory capacity, multimedia design principles (e.g., those derived from CTML) aim to manage processing demands and reduce unnecessary cognitive load. A. D. Baddeley and Hitch’s (1974) multicomponent model includes the phonological loop, which maintains and rehearses verbal and auditory information; the visuospatial sketchpad, which maintains and manipulates visuospatial information; and the central executive, which allocates attention and coordinates processing. Later refinements added the episodic buffer, a limited-capacity store that binds information from different sources and provides an interface between working memory and long-term memory (A. Baddeley, 2000). Learning may be hindered when instructional materials impose processing demands exceeding available resources.

Since working memory capacity is typically treated as an individual-difference measure, cognitive load theory (CLT) provides a framework guiding material design under these constraints. In the CLT framework, cognitive processing comprises three types: intrinsic, extraneous, and germane (Sweller, 2010). Intrinsic cognitive load reflects the inherent difficulty of information, which varies individually due to prior knowledge, whereas extraneous cognitive load results from poorly designed environments that hinder schema construction or automation (Paas et al., 2004; Kalyuga, 2011; Mayer, 2011). Germane cognitive load allows working memory to activate schemas and integrate information into long-term memory (Sweller, 2010). The relationship between cognitive load and mental processing is not straightforward, as working memory operates under strict limitations and different forms of cognitive load interact dynamically. Because intrinsic and extraneous cognitive load are additive, changes in task complexity or instructional design can shift total load imposed on learners (Leppink et al., 2015).

To date, no prior studies have examined cognitive load and working memory together in video podcasts. Research in adjacent fields, such as video lectures, can inform expectations when CTML design principles are applied. A systematic review on cognitive load research in multimedia learning found that extraneous cognitive load was studied more often than intrinsic cognitive load, germane cognitive load was rarely examined, and almost half of studies used a single cognitive load concept with no type categorization (Mutlu-Bayraktar et al., 2019). The review found that the modality principle (using spoken words rather than redundant on-screen text), signaling principle (directing learner attention), and coherence principle (removing extraneous information such as irrelevant words, pictures, sounds, or decorative elements) help manage cognitive load. Similarly, Aalioui et al. (2022) found that the segmenting principle (dividing longer lectures into shorter units) reduced cognitive load compared to longer, continuous videos.

Although CTML principles have demonstrated that such principles reduce cognitive load, working memory, a fundamental component of cognitive load theory, has been rarely conceptualized or measured in multimedia learning (Anmarkrud et al., 2019; Mutlu-Bayraktar et al., 2019). When assessed, evidence suggests a complicated relationship between design elements and outcomes. For example, Zhang et al. (2022) found learners with lower working memory capacity benefit more from instructor presence or structuring cues in instructional videos, whereas higher-capacity learners are less affected. Conversely, Skuballa et al. (2012) found that signaling and verbal instructions impacted students with low and high working memory differently. Learners with low working memory showed poorer performance when verbal instructions accompanied animations, while high working memory learners performed worse with cueing strategies. Despite working memory and cognitive load being central design considerations for video-based studies, most do not explicitly measure or analyze working memory capacity as a predictor or moderator (Brame, 2017).

### 2.2. Situational Interest

Situational interest (SI) is a context-dependent state that emerges in response to particular tasks or environmental features and is characterized by attentional and affective reactions, rather than stable topic preferences (Hidi & Renninger, 2006). It is tightly bound to the learning situation, including how content is presented, the types of examples used, or how relevant materials appear in the moment (Hidi & Renninger, 2006; Linnenbrink-Garcia et al., 2010). Research using factor analysis and longitudinal regression found that although individual interest and SI may overlap, they are distinct constructs, with SI consisting of three factors: triggered-SI, maintained-SI-feeling, and maintained-SI-value (Linnenbrink-Garcia et al., 2010). Triggered-SI involves attention directed toward classroom material presentation. In contrast, maintained-SI is a sustained form of interest connected to learners’ perceived meaning of the content. Maintained-SI-feeling represents intrinsic enjoyment and positive affect experienced during engagement with the material, while maintained-SI-value represents learners’ reflective judgments of content relevance and usefulness.

To date, no studies have measured situational interest with video podcasts. However, studies examining presentation design and video lectures in relation to situational interest show mixed results, likely due to differences in how situational interest has been defined and measured. Some use the Linnenbrink-Garcia et al. (2010) scale dividing situational interest into triggered-SI, maintained-SI-feeling, and maintained-SI-value; others use variations with similar components (triggered-SI, maintained-SI) or a single situational interest scale. Using the three subscales by Linnenbrink-Garcia et al. (2010), Bland et al. (2024) found that redesigning PowerPoint slides with CTML principles (segmenting) and other design strategies significantly increased situational interest across all three scales compared to the control condition. Similarly, using an early version including triggered-SI and combined maintained-SI (Hidi & Renninger, 2006), Endres et al. (2024) examined situational interest in a 2 × 2 video-instruction design crossing emotional versus neutral audiovisual design with narrative versus expository framing. Emotional audiovisual design significantly increased triggered-SI relative to neutral design, and narrative framing enhanced triggered-SI development into maintained-SI. Maintained-SI was positively associated with sustained learning, even though audiovisual design showed no direct performance effect, indicating that emotional design and narrative framing primarily influence learning by shaping learners’ interest during video instruction.

Studies measuring situational interest as a single concept revealed nuanced findings. Wang et al. (2020) examined video instructor presence across instructional topics classified as easy or difficult. Results indicated that situational interest was significantly higher when an instructor was present, regardless of topic difficulty, suggesting that instructional design features such as instructor presence may play a more critical role in fostering learner interest than topic difficulty alone. However, using a single situational interest construct, Anttonen et al. (2024) found no significant differences between storified and non-storified instructional conditions when both adhered to CTML principles, including segmentation, signaling, and personalization). The inclusion of additional narrative elements, such as plot, setting, and recurring story components, did not significantly influence situational interest. Taken together, these findings suggest that additive contextual features may have limited impact on situational interest when foundational instructional design principles are consistently applied.

### 2.3. Elaborative and Seductive Details

Additive contextual features such as elaborative details or seductive details can alter the learning context, though they have different effects. Elaborative details assist learners with cognitive processing of information. Elaboration is grounded in the levels-of-processing framework, which suggests learners encode new information by linking it to existing knowledge structures through meaningful associations, images, or related knowledge (Craik & Lockhart, 1972). Elaboration enriches information through deeper semantic analysis and connections to prior knowledge, enabling relational mapping. Later literature refers to these meaning-based enrichment processes as elaborative encoding. Craik and Tulving’s (1975) findings showed that memory benefits when learners engage in deeper, semantically oriented processing. Elaborative encoding encompasses various techniques helping learners link new information with existing knowledge. For example, Jaeger et al. (2025) explored learner-generated elaborative strategies such as imagining interactive scenes or generating sentences integrating paired concepts.

In multimedia environments, elaborative encoding concepts are often termed generative processing, which refers to learners’ active efforts to select, organize, and integrate information with prior knowledge (Mayer, 2020). Instructional features prompting explanation, imagery, or relational thinking can foster generative processing, whereas added details not supporting meaningful integration may divert cognitive resources and hinder learning (Fiorella & Mayer, 2016). Fiorella (2023) reviewed experimental studies demonstrating that learner-generated activities such as explaining, imagining, and drawing consistently improved learning and transfer compared to passive study. These benefits occur because generative activities prompt learners to actively organize and integrate information with prior knowledge, particularly when tasks align with instructional goals and do not impose excessive extraneous cognitive load.

Analogies are another type of elaborative encoding supporting generative processing by helping learners map new ideas onto familiar knowledge structures. Analogical explanations help learners identify structural correspondences between something known (e.g., water flow in pipes) and information being learned (e.g., electric current), thereby encouraging relational thinking and integration with prior knowledge rather than pure memorization (Glynn, 2008). When clearly mapped and aligned with core concepts, analogies can make abstract processes more concrete and improve conceptual understanding (Gentner & Markman, 1997). However, poorly designed or overly complex analogies may increase extraneous cognitive load or foster misconceptions by introducing irrelevant relationships or excessive relational demands (Harrison & Coll, 2008). Effective multimedia analogies should be carefully selected, visually supported, and accompanied by explicit clarification of their limitations to harness their generative potential without overloading working memory (Petchey et al., 2023).

Conversely, seductive details are interesting but instructionally irrelevant additions that may interfere with coherent mental model construction (Mayer, 2020). Seductive details are commonly framed within the coherence principle, which suggests that extraneous words, pictures, and sounds hinder learning by distracting limited cognitive resources from selecting, organizing, and integrating core content (Mayer, 2020). Several reasons have been proposed for why seductive details impair learning. Seductive details may divert attention toward irrelevant information, disrupt coherence by interfering with essential material organization, and consume cognitive resources by processing irrelevant information, thereby increasing extraneous cognitive load (Harp & Mayer, 1998; Rey, 2012; Mayer, 2020). Meta-analytic evidence indicates that seductive details can undermine learning outcomes (Rey, 2012; Sundararajan & Adesope, 2020).

### 2.4. Research Questions

This exploratory study examines how individual differences in working memory capacity relate to video podcasts that include different types of additive contextual features, such as elaborative and seductive details. This study investigates how these additive features influence cognitive load, situational interest, and learning outcomes with and without working memory as a covariate. Although video podcasts are widely used in higher education, additive contextual features and core cognitive constructs such as working memory, cognitive load, and situational interest, have rarely been examined together in multimedia environments. Accordingly, this study aims to clarify whether observed effects in video podcast learning reflect individual differences in cognitive capacity, instructional design features, or a combination of elements. The research addresses the following questions:

RQ1a: To what extent does working memory capacity affect learners’ cognitive load when studying video podcasts with different additive contextual features (elaborative vs. seductive details)?

RQ1b: To what extent do additive contextual features affect learners’ cognitive load when studying with video podcasts?

RQ2a: How does working memory capacity affect learners’ situational interest when studying video podcasts with different additive contextual features?

RQ2b: How do additive contextual features affect learners’ situational interest when studying with video podcasts?

RQ3a: To what extent does working memory capacity affect learners’ learning outcomes (free recall and transfer) when studying video podcasts with different additive contextual features?

RQ3b: To what extent do additive contextual features affect learning outcomes (free recall and transfer) when studying with video podcasts?

Interview data will be used to interpret and contextualize the quantitative findings by providing additional insight into how learners experienced and made sense of the different additive contextual features during video podcast study.

## 3. Materials and Methods

### 3.1. Research Design and Participants

A between-subjects experiment examined outcomes of multimedia educational video podcasts with a standard script compared to those with elaborative or seductive details. Data were collected in a university computer lab equipped with monitors and headphones. Before beginning the experiment, students were informed of their participation rights orally in English and allowed to ask questions. Additionally, participants were provided a computerized consent form in English and Korean that they had to agree to before continuing. The experiment lasted no more than 40 min.

The study comprised 62 students attending a foreign-language university in Seoul, South Korea. The sample consisted of 16 males and 46 females. Academic levels included one freshman, 14 sophomores, 19 juniors, and 28 seniors. Students were advanced non-native English speakers majoring or double majoring in English-related fields such as English translation, English linguistics, international finance, or international studies. The average age was 22.94 years (SD = 2.64). Participants were randomly assigned to one of three conditions: control (*n* = 23), elaborative (*n* = 20), and seductive details (*n* = 19). Five students (elaborative = 2, seductive details = 3) did not complete the study and were removed from the analysis.

An a priori power analysis was conducted in G*Power 3.1 for a one-way between-subjects ANOVA with three conditions (α = 0.05, 1 − β = 0.80). Assuming a medium effect size (f = 0.25), the required total sample size was N = 156. Due to environmental constraints, the final sample consisted of 62 participants (control *n* = 23, elaborative *n* = 20, seductive details *n* = 19). A sensitivity analysis was conducted in G*Power using the achieved sample size and group structure (α = 0.05, 1 − β = 0.80, 3 groups), which indicated that the study had 80% power to detect effects of f = 0.40 or larger.

### 3.2. Instructional Content and Multimedia Design

The research topic focused on lightning formation and used the 287-word script from Moreno and Mayer (1999). Although Google’s NotebookLM can convert scripts into podcasts, the generative AI interface does not allow users to tailor content to their needs. Therefore, Google Gemini 2.5 was used to create a two-person podcast script that included a detailed introduction and sign-off that was standard across all conditions. The host served as an interviewer, presenting the introduction and sign-off and asking questions or making brief statements to mimic an educational podcast conversation. The co-host served as a weather expert, presenting the information on lightning formation from the Moreno and Mayer (1999) script.

The AI-created script restated some of the original information using different sentences to convey the same ideas, so the authors reviewed and altered the scripts to ensure that key sentences from the original script were embedded in the AI-created versions. Following this, the researchers selected seven key ideas from the script to which either elaborative details or seductive details were added. The added details corresponded to the same key idea in each script to create balance and minimize confounding variables related to the information being presented. The control condition included only the co-host presenting the original information, with no additional details.

After finalizing the scripts, AI voices from elevenlabs.io were used to present the information. The voice of Jane (British English, professional audiobook reader) was used for the host, while Corey (American English, friendly, clear, and approachable) voiced the co-host. Audio was recorded according to the scripted turn-taking structure. To maintain recording stability and voice consistency, longer dialogue turns were split into two or more segments and merged into a single utterance during post-production using Camtasia 2024. Similarly, 15 static images depicting lightning formation were created in Adobe Illustrator 25.3 (Davis et al., 2024) based on Mayer and Moreno (2002). In all conditions, each static illustration appeared with its corresponding information and disappeared upon completion of that information. Since all conditions contained the same core language from the original script, each condition was exposed to the visual aids for the same duration to maintain balance across conditions. Each condition included a 5 s title card with the logo and title of the podcast “Weather Wonders” with thunder and rain sounds fading in the background. The control condition video was 3:51 min in length, the elaborative condition was 5:50 min, and the seductive details condition was 6:23 min.

### 3.3. Instruments

#### Demographic Survey and Prior Knowledge Test

After agreeing to informed consent, participants provided basic demographic information including year in university (freshman, sophomore, junior, senior, other), international age (as distinct from the Korean age system), native language (Korean, English, Russian, etc.), and major (English linguistics, English for International Conferences and Communication, International Business, etc.). This information was used to assess whether any demographic characteristics affected the outcome measures.

After providing demographic information, participants completed a prior knowledge assessment to determine whether groups differed in prior weather knowledge. The prior knowledge assessment utilized the standard assessment for this topic by Moreno and Mayer (1999). The assessment consisted of seven yes-no questions and one Likert scale question ranging from 1 (very little) to 5 (very much). The yes-no questions measured basic weather knowledge and were awarded one point for each positive answer, while the Likert scale question assessed general understanding of weather. Prior knowledge did not differ significantly across conditions (*p* = .836).

### 3.4. Cognitive Load

After listening to the video podcast, participants completed a cognitive load questionnaire adapted from Leppink et al. (2013) to measure intrinsic (3 items), extraneous (3 items), and germane cognitive load (4 items). Each item was rated on a 10-point Likert scale ranging from 1 (strongly disagree) to 10 (strongly agree). Internal consistency was measured using Cronbach’s alpha: intrinsic cognitive load (α = 0.87), extraneous cognitive load (α = 0.85), and germane cognitive load (α = 0.93), indicating good to excellent reliability.

### 3.5. Situational Interest

Learners’ situational interest in educational video podcasts was measured using an adapted version of the Situational Interest Survey developed by Linnenbrink-Garcia et al. (2010). The adapted survey consisted of 14 items rated on a 7-point Likert scale ranging from 1 (strongly disagree) to 7 (strongly agree). The items were organized into three subscales: triggered situational interest (5 items), maintained situational interest-feeling (5 items), and maintained situational interest-value (4 items). Triggered situational interest assessed affective reactions to the educational video podcast, maintained situational interest-feeling captured affective engagement with the content, and maintained situational interest-value focused on the perceived importance and usefulness of the content. Three items in triggered situational interest were reverse scored (“I don’t like the podcast very much,” “The podcast in this study isn’t very interesting,” and “The podcast in this study really seems to drag on forever”), and one item from maintained situational interest-feeling was reverse scored (“To be honest, I just don’t find the podcast topic interesting”). Internal consistency was measured using Cronbach’s alpha: triggered situational interest (α = 0.73), maintained situational interest-feeling (α = 0.92), and maintained situational interest-value (α = 0.83), indicating acceptable to excellent reliability.

### 3.6. Digit Span

A digit span task was administered through PsyToolKit, a web-based platform for creating and running cognitive-psychological experiments and surveys, to measure students’ short-term verbal working memory. The digit span test requires participants to reproduce sequences of digits that increase in length over trials. Participants were first presented with instructions explaining that the system would present a series of digits that they would need to recall in the same order. Each trial consisted of a sequence of digits ranging from 0 to 9 that were presented one at a time in the center of the screen at a fixed rate. Participants then attempted to recall the sequence of numbers in the original order on another screen.

The sequence started with two numbers and only increased by one digit in length when the participant correctly reproduced the sequence on at least two trials. The task ended when participants failed to reproduce the sequence twice at the same length. Each participant’s maximum length was recorded as an individual digit span score. Working memory capacity was indexed by the maximum digit span score, defined as the longest sequence of digits correctly recalled. This score was entered as a covariate in MANCOVA and ANCOVA analyses to control for individual differences in working memory.

### 3.7. Free Recall and Transfer Learning Outcomes

Free recall was measured using a single open-ended prompt asking participants to explain the process of lightning formation. Responses were evaluated using a predefined scoring rubric consisting of 19 idea units from Moreno and Mayer (1999), with participants receiving one point for each correctly recalled unit. Two raters independently scored all responses, achieving substantial agreement (κ = 0.66; Cohen, 1960). Inconsistencies were resolved through consensus discussions involving a third scorer.

Participants then completed a transfer test comprising four open-ended questions designed to assess deeper understanding. Scoring followed the response guidelines established by Moreno and Mayer (1999), with a total possible score of 12 points distributed across the four questions. Two independent raters scored the responses, and disagreements were resolved through discussion with a third party. Interrater reliability was substantial (κ = 0.75; Cohen, 1960).

### 3.8. Interviews

To further explore participant perspectives on elaborative and seductive details within the educational video podcast, interested individuals provided their email addresses to schedule individual interviews for the following week. Interviews were conducted by a professor from another university who is fluent in both Korean and English and was unknown to the participants. Each interview lasted approximately 20 min and focused on the participant’s interest in the video podcast, how cognitively difficult the information was, the learning experience, and their general experience learning with an educational video podcast. Eleven participants from the control condition (*n* = 3), the elaborative details condition (*n* = 4), and the seductive details condition (*n* = 4) were originally contacted for interviews. However, four never returned the first or follow-up emails, and one scheduled an interview but did not attend and did not return a follow-up email.

## 4. Results

### 4.1. Cognitive Load

To test whether working memory affected cognitive load, ANCOVAs were conducted with working memory as a covariate. Results indicated that working memory did not affect intrinsic cognitive load (F(2, 58) = 0.386, *p* = .682, partial η^2^ = 0.013), extraneous cognitive load (F(2, 58) = 0.357, *p* = .701, partial η^2^ = 0.012), or germane cognitive load (F(2, 58) = 0.207, *p* = .813, partial η^2^ = 0.007). See Table 1 for cognitive load means and standard deviations.

To assess whether additive contextual features affected students perceived cognitive load, an ANOVA assessed student ratings on intrinsic cognitive load, extraneous cognitive load, and germane cognitive load. Results indicated no significant differences between conditions for intrinsic cognitive load (F(2, 59) = 0.514, *p* = .601, partial η^2^ = 0.017), extraneous cognitive load (F(2, 59) = 0.491, *p* = .615, partial η^2^ = 0.016), or germane cognitive load (F(2, 59) = 0.200, *p* = .819, partial η^2^ = 0.007). 

### 4.2. Situational Interest

A MANCOVA was performed using working memory as a covariate. No significant differences were found, F(6, 112) = 0.476, *p* = .827; Wilks’ λ = 0.951, partial η^2^ = 0.025. This indicates that the working memory covariate did not reveal additional treatment effects, as the multivariate effect size decreased from the initial MANOVA model. See Table 2 for the means and standard deviations of situational interest.

To measure the effects of additive contextual features on situational interest, a MANOVA was conducted on the three subscales of triggered situational interest, maintained situational interest-feeling, and maintained situational interest-value. Box’s M (16.94) indicated no violation of homogeneity (*p* = .207). A Wilks’ Lambda test found no significant differences between conditions, F(6, 114) = 0.770, *p* = 0.595; Wilks’ λ = 0.924, partial η^2^ = 0.039. See Table 2 for all means and standard deviations for situational interest.

### 4.3. Free Recall

An ANCOVA was conducted to test whether working memory affected free recall across the three conditions. The interaction between condition and working memory was not significant, F(2, 32) = 0.235, *p* = .792, partial η^2^ = 0.014. Controlling for working memory, free recall did not differ significantly across conditions, F(2, 32) = 0.678, *p* = .515, partial η^2^ = 0.041, and working memory was not a significant covariate, F(1, 32) = 0.018, *p* = .893, partial η^2^ = 0.001. Estimated marginal means adjusted at the sample mean of working memory (WM = 4.29) suggested higher free recall in the control condition (Madj = 5.89) than in the elaborative (Madj = 2.47) and seductive details (Madj = 2.51) conditions. However, Bonferroni-adjusted pairwise comparisons were not statistically significant (control vs. elaborative: *p* = .059; control vs. seductive details: *p* = .092; elaborative vs. seductive details: *p* = 1.000).

An ANOVA was performed to assess whether additive contextual features affected free recall. The ANOVA revealed a significant difference between conditions, F(2, 59) = 6.18, *p* = .004, partial η^2^ = 0.173. Homogeneity of variances was violated, as indicated by Levene’s test (*p* = .028); therefore, Games-Howell post hoc tests were used. Pairwise effect sizes are reported as Hedges’ g to account for unequal variances. Games-Howell tests indicated that the control condition scored significantly higher than the seductive detail condition (*p* = .004, g = 1.03). The difference between the control and elaborative detail conditions did not reach significance (*p* = .077, g = 0.73), nor did the difference between the elaborative and seductive detail conditions (*p* = .497, g = 0.30).

### 4.4. Transfer

An ANCOVA was conducted to test whether working memory affected transfer across the three conditions. The interaction between condition and working memory was not significant, F(2, 32) = 1.667, *p* = .205, partial η^2^ = 0.094. Controlling for working memory, transfer did not differ significantly across conditions, F(2, 32) = 1.814, *p* = .179, partial η^2^ = 0.102, and working memory was not a significant covariate, F(1, 32) = 2.072, *p* = .160, partial η^2^ = 0.061. Estimated marginal means adjusted at the sample mean of working memory (WM = 4.29) were Madj = 0.901 for the control condition, Madj = 0.646 for the elaborative details condition, and Madj = 0.822 for the seductive details condition. Bonferroni-adjusted pairwise comparisons indicated no significant differences between conditions, with all *p*-values at 1.000.

An ANOVA was performed to assess whether additive contextual features affected transfer. The ANOVA did not reveal a significant difference between conditions, F(2, 59) = 0.143, *p* = .867, partial η^2^ = 0.005. Levene’s test indicated that the homogeneity of variances was not violated (*p* = .064). See Table 3 for all the means and standard deviations for free recall and transfer.

### 4.5. Interviews

Six students participated in the follow-up interview phase. No course credit, grade-related advantages, or material compensation were offered. Before each session, participants were fully informed of the study’s purpose and consent procedures, and all agreed to participate under these conditions. The interviews were conducted and recorded via Zoom, with each lasting approximately 20 min.

Interview participants represented a diverse mix of nationalities, academic levels, and linguistic backgrounds. The control group included one Korean senior (S1). The seductive details group consisted of two seniors, one French (S2) and one Swedish (S3). The elaborative details group comprised three students: a German junior (S4), an Uzbek sophomore (S5), and a Korean senior (S6). In accordance with the interview protocol, all sessions were used solely for research purposes. Participants were assured complete anonymity, and their identities were protected by referring to them using coded identifiers. See Table 4 for participants and conditions.

#### 4.5.1. Interview Data Analysis

Interview data were analyzed using Braun and Clarke’s (2006) thematic analysis, enabling detailed interpretation of participants’ experiences across the three conditions. The analysis began with multiple close readings of transcripts to gain thorough understanding of students’ responses. During this stage, preliminary codes were generated to highlight meaningful ideas related to interest, comprehension, cognitive load, and perceived usefulness of visual and auditory features. These codes were gradually organized into broader, coherent categories by comparing patterns across participants from the control, seductive details, and elaborative details groups. As the analysis progressed, themes were refined to capture nuances accurately and ensure each theme was supported by clear, consistent evidence. The final themes reflected several recurring ideas, including the importance of visual aids in supporting comprehension, the value of conversational dialogue over monologic explanation, and the challenges posed by scientific terminology. This iterative process provided a holistic view of how students made sense of the instructional video podcast and how each condition influenced their learning experience.

#### 4.5.2. Findings from the Interviews

##### Interest

Analysis of interview transcripts revealed two major themes pertaining to interest. The most common theme across all three conditions was the use of visuals within the educational video podcast. Every participant interviewed highlighted that visuals were a key element in generating interest. However, most referred to visuals as a design feature that helped maintain their interest and supported understanding of the content. S4 noted that the visuals directly supported her understanding: “The video explained it pretty well, I think, and the drawings really helped make it more understandable.” Likewise, S3 expressed a similar view: “It was good with the images and simple drawing, so it made it very easy to understand.” Other students suggested that visuals were a key aspect of their interest but expressed concerns that the visuals were not consistent or there were not enough visuals. S6 stated that while visuals were helpful, they could cause confusion: “The visuals were very helpful. That said, the images appeared and disappeared intermittently, only during certain parts. So it was a bit confusing to know when to focus.” S2 indicated that more visual aids should have been included: “For a six-minute video…there should be more visuals with it.” However, S1 in the control condition expressed that the entertainment value was limited by the constraints of the pictures themselves. S1 highlighted: “And it’s showing like how it’s working with just simple pictures… so this captured my attention well.” These comments highlight that design features and situational interest are linked. Specifically, the qualitative data suggest that while visuals serve as a powerful hook for interest, the intermittent appearance of visuals can create issues. For educational video podcasts, this provides support for the modality principle, suggesting that combining spoken words with visuals can increase attention and facilitate learning.

Another design theme that increased interest for some students was the use of two presenters. Only students in the elaborative details and control conditions mentioned the conversational style presentation. S4 and S5 in the elaborative details condition found the conversation style engaging. S4 highlights: “I liked that the two speakers kept going back and forth. I liked how the female speaker re-asked some questions.” S5 stated: “In the podcast where two people discuss it (lightning formation), like an interview, makes this podcast interesting.” S1 in the control condition found the interview-style presentation interesting but noted that the narration could have been more engaging and lively rather than strictly educational in tone. He suggested: “It wasn’t the kind of podcast where the hosts spoke in a lively or humorous way; it was clearly and educational tone.”

Overall, interest appeared to stem primarily from the design of the video podcast rather than the content itself. Of the six participants, only S5 mentioned that the topic increased interest. This suggests that interest was more design-based than topic-driven. Participants consistently framed their interest in terms of how material was presented, primarily the use of visuals and the conversational structure of the narration, rather than the subject matter itself. This finding suggests that instructional design choices play a critical role in eliciting and sustaining learner interest in multimedia environments.

##### Learning

Analysis of interview transcripts revealed patterns indicating that participants in both the seductive details and elaborative details conditions experienced difficulty listening for essential information about lightning formation.

Interview participants in the seductive details condition reported confusion about core scientific mechanisms. S2 reported that the seductive details made it more difficult to understand: “It started by saying that heat would go up and the cold would do down, and that it creates ice, but that the clouds were full of dirt and ashes. It was hard to understand the main point.” This suggests that the additional details diverted her attention from key concepts. Similarly, S3 remarked that it contained too much in one section, suggesting that the seductive additions increased cognitive load: “For example, the positive and negative charges were interesting, but it was too much at once.” These findings suggest that seductive details distracted learners from the primary explanatory sequence and impaired their recall of essential steps in lightning formation.

Interview participants in the elaborative details condition displayed similar challenges, despite elaborative details being designed to facilitate comprehension. S5 stated that the explanation made difficult to follow and contributed to a sense of information overload. He stated: “I think the podcast was interesting, but sometimes it had a lot of topics, making it a little bit complicated.” This suggests the elaborative verbal information was at times overwhelming. Similarly, S6 expressed confusion about the formation and movement of positive and negative charges inside the cloud: “I couldn’t quite grasp how positive and negative charges are formed inside the cloud. Without images, I don’t think I’d have understood anything.” This indicates that elaborative content did not promote deeper understanding. S4’s interview further reinforced this pattern: although she found the drawings helpful, she reported: “I thought it was harder to follow when there wasn’t a drawing. There should be more drawings or animations throughout the whole video,” suggesting that pictures were more important than elaborative verbal explanations for recall.

In contrast, the control condition did not exhibit this pattern of distraction caused by additional information. S1 attributed her difficulties to unfamiliar terminology and limited background knowledge rather than excessive or unnecessary content. She stated: “There was a lot of unfamiliar content, I did feel it was quite dense. The information wasn’t necessarily difficult, but I had to stay focused the whole time to understand it.”

Overall, interview results suggest that the additive contextual features of elaborative details and seductive details added complexity to the content. However, the control condition did have significantly higher free recall than the seductive details condition. Consistent with previous literature, seductive details participants were distracted by non-essential, attention-grabbing details, while learners in the elaborative details condition might have struggled with density and complexity; however, the lack of additional details may have allowed for more generative processing when freely recalling information. These qualitative insights suggest that additional information may affect the ability to freely recall information but might not affect cognitive load, situational interest, and transfer of information.

## 5. Discussion

Routinely, research questions would be addressed individually, but given how working memory capacity is intertwined with perceived cognitive load and CTML, this discussion is presented by outcome domain. Before interpreting these findings, an important methodological limitation should be noted. The final sample (N = 62) was smaller than the a priori target (N = 156), providing adequate power only to detect effects of f = 0.40 or larger. Accordingly, findings for cognitive load, situational interest, and transfer should be interpreted cautiously, as smaller but meaningful effects may not have been detected. Where these null results are consistent with theoretical expectations, the interpretations should be considered tentative and in need of replication with larger samples.

### 5.1. Cognitive Processing

Results indicated that working memory capacity and cognitive load were not significant predictors across conditions. Because working memory did not emerge as a meaningful covariate across measures and cognitive load did not differ between conditions, these findings may suggest that instructional design features constrained cognitive processing demands, though this interpretation should be treated cautiously given the underpowered sample. From a CLT and CTML perspective, this is consistent with well-designed multimedia instruction offsetting working memory limitations and reducing extraneous processing (Mayer, 2020; Sweller et al., 2019). The presentation of narrated information and visual illustrations aligns with the modality principle, which suggests learning is enhanced when verbal and pictorial information are presented through complementary channels that help build stronger mental models (Mayer, 2020).

The absence of differences in cognitive load between conditions reinforces this interpretation. Despite the inclusion of a seductive details condition, intrinsic, extraneous, and germane cognitive load did not differ significantly across conditions. From a cognitive load perspective, seductive details are expected to increase extraneous cognitive load by drawing attention and processing away from essential content (Harp & Mayer, 1998; Mayer, 2020). However, the absence of significant differences in quantitative data warrants nuanced exploration based on qualitative data. Interview data revealed that students in the elaborative details and seductive details conditions felt overwhelmed at times with scientific terminology and additional information. Although quantitative and qualitative data appear contradictory, the qualitative data revealed that many students found visual supports key to understanding the information. Because working memory and cognitive load are intertwined, the design of the video podcast according to the modality principle and dual-channel processing may have prevented students from experiencing significant extraneous load, as offloading verbal narration to the auditory channel may have freed sufficient visual channel capacity to maintain coherent mental models despite the additive details (Mayer & Moreno, 2003). Students routinely mentioned in interviews the need for visual information to understand the narration better, and as advanced-level second language users, students may have relied heavily on the visual channel to maintain coherent mental models. Future research may explore elaborative details and seductive details without illustrations to understand if these conditions affect working memory or cognitive load.

### 5.2. Situational Interest

Results for situational interest found no significant differences between conditions. These results align with previous research finding that additional narrative features did not significantly influence situational interest (Anttonen et al., 2024). In other words, designing the video podcast with elaborative or seductive details did not significantly increase interest in the current sample, though these findings should be interpreted cautiously given the limited statistical power. Although there were no significant findings, interview data supported previous research suggesting that content presentation, relevancy of materials, and examples could factor into situational interest (Hidi & Renninger, 2006; Linnenbrink-Garcia et al., 2010). When asked about their interest in the video podcasts, almost all students mentioned that illustrations increased their interest. Similarly, another design aspect that increased situational interest was information being presented as a conversation between two speakers. Since design features were the same across videos, this may help explain why no significant differences emerged between conditions. Further research should explore which design features increase student interest, such as static images versus animations, or conversational versus single-narrator formats. Although video podcasts are not new, further exploration of multimedia principles applied to educational video podcasts is needed.

### 5.3. Learning Outcomes

Results for free recall indicated that students in the control condition outperformed those in the seductive details condition, while no other differences between conditions were observed. However, when working memory was included as a covariate, these differences were no longer significant. For transfer, no significant differences were found between conditions, with or without working memory as a covariate. Together, these findings tentatively suggest that added contextual information provided no measurable benefits on learning outcomes.

The significantly higher recall in the control condition over the seductive details condition aligns with prior findings that seductive details can impair recall by diverting attention to interesting but nonessential information, thereby disrupting the coherence of learners’ mental models (Harp & Mayer, 1998; Rey, 2012; Sundararajan & Adesope, 2020). In contrast, the absence of differences between the control and elaborative details conditions for free recall may suggest that not all additional information is equally detrimental. When added information is perceived as supportive or explanatory, it may not disrupt recall to the same extent as seductive details. However, when working memory capacity was included as a covariate, free-recall differences between conditions were no longer significant, and working memory itself did not emerge as a significant predictor. This suggests that the observed advantage of the control condition over the seductive details condition may reflect design-related differences in how information was selected and organized, rather than learners’ short-term storage capacity. In other words, seductive details may have interfered with the consolidation and retrieval of core idea units regardless of measured individual differences in working memory.

The absence of significant differences for transfer across instructional conditions, with or without working memory as a covariate, suggests that the manipulations employed were insufficient to influence learners’ ability to apply knowledge to novel problems. From a cognitive load theory perspective, transfer is most likely to occur when instruction reduces extraneous cognitive load and promotes learning-relevant processing without exceeding working-memory capacity (Sweller et al., 2019). Variations in surface-level features such as additive contextual information often yield limited effects on transfer outcomes.

Overall, within the constraints of the current sample, added contextual features, particularly elaborative details, did not provide benefits for learning. Participants in both the elaborative details and seductive details conditions described the additional information as unnecessary or overwhelming, especially combined with unfamiliar scientific terminology. Although cognitive load measures did not signal that extraneous cognitive load was a factor, interview data provided evidence that students were managing more complexity in the additive contextual features conditions than the control condition, which only struggled with scientific terminology. Despite these findings, the potential of additive contextual features like elaborative details warrants further investigation. Interview data revealed that students in the elaborative details condition occasionally struggled to distinguish the main points. This suggests that future research should examine the role of verbal signaling to help learners navigate complex content and prioritize salient information more effectively.

### 5.4. Limitations

Several limitations should be acknowledged, and the findings reported here should be interpreted with appropriate caution given the exploratory nature of the study and the constraints of the sample. First, the convenience sample included only 62 participants, providing 80% power to detect effects of f = 0.40 or larger. Small to medium effect sizes may not have been detected in cognitive load or situational interest across conditions. Likewise, smaller but meaningful effects of working memory capacity or additive contextual features may not have been detected. The present findings should therefore be interpreted cautiously, and replication with adequately powered samples is encouraged before drawing generalizable conclusions.

Second, students in this research were advanced second language users of English. Although students participate in English-only lectures and speak English at near-native levels, the content included scientific terminology that might have been difficult for students studying the humanities. Processing academic content in a second language imposes additional cognitive demands that may have led students to rely more heavily on the visual channel to maintain comprehension, potentially contributing to the stability of cognitive load across conditions. Therefore, these results may not be generalizable to native English speakers or lower-level non-native speaking populations.

Finally, the working memory capacity assessment was delivered through a digit span task, which assesses only short-term verbal storage. The digit span task was selected because the experimental manipulation consisted of additive verbal content delivered through narration, making the phonological loop the most directly implicated component of working memory in this study. Although this research included limited visuospatial elements such as illustrations, future assessments should include both verbal and visuospatial components of working memory capacity, which might reveal different relationships between working memory and other measures when learning with video podcasts.

## 6. Conclusions

This study examined the role of working memory capacity and additive contextual features in educational video podcasts, focusing on cognitive load, situational interest, and learning outcomes. Overall, working memory did not emerge as a meaningful predictor across measures, suggesting that the combination of narration and closely aligned visuals constrained processing demands, though this interpretation requires replication with larger samples. Similarly, elaborative and seductive details did not significantly affect perceived cognitive load or situational interest, tentatively suggesting that within a well-structured multimedia environment, additive contextual features may exert relatively subtle effects on learners’ subjective experiences.

Differences were most evident for free recall, with students in the control condition outperforming those in the seductive-details condition, consistent with prior research on coherence and seductive details condition. However, these differences became non-significant when working memory was included as a covariate, and no differences were observed for transfer. Together, these findings tentatively suggest that additive contextual features are more likely to influence surface-level recall than deeper conceptual transfer.

Qualitative findings further informed these results by revealing how learners experienced the added details. Participants frequently described seductive details and elaborative details as increasing information density, alongside scientific language that affected their perceived ability to comprehend the materials. Across conditions, learners consistently emphasized the importance of visuals for understanding, highlighting the stabilizing role of core multimedia design features.

Overall, this preliminary mixed-methods evidence suggests that in carefully designed educational video podcasts, instructional coherence and visual support may play a more central role than working memory capacity in shaping learning outcomes, though these findings should be interpreted cautiously and replicated with larger samples before broader conclusions are drawn. Based on these preliminary findings, designers of educational video podcasts may benefit from prioritizing narration aligned with supportive visuals, though further research is needed to confirm these recommendations. Future research should examine multimedia design features and use broader working memory measures to identify the conditions under which video podcasts best support learning.

## Figures and Tables

**Table 1:** Means and Standard Deviations of Cognitive Load.

	Intrinsic Cognitive Load	Extraneous Cognitive Load	Germane Cognitive Load
	N (SD)	N (SD)	N (SD)
Control Condition	11.30 (4.20)	8.04 (4.27)	27.35 (7.45)
Elaborative Details Condition	12.90 (6.67)	9.60 (5.98)	27.85 (6.93)
Seductive Details Condition	12.90 (7.02)	9.05 (5.49)	26.26 (9.92)

**Table 2:** Means and Standard Deviations of Situational Interest.

	Triggered Situational Interest	Maintained Situational Interest—Feeling	Maintained Situational Interest—Value
	N (SD)	N (SD)	N (SD)
Control Condition	24.22 (5.05)	21.26 (6.45)	17.39 (4.10)
Elaborative Details Condition	23.40 (5.35)	18.50 (7.57)	17.50 (4.56)
Seductive Details Condition	21.84 (7.23)	19.16 (7.57)	19.00 (5.55)

**Table 3:** Means and Standard Deviations for Free Recall and Transfer Scores.

	Free Recall	Transfer
	N (SD)	N (SD)
Control Condition	5.52 (3.99)	0.70 (0.93)
Elaborative Details Condition	3.10 (3.11)	0.60 (0.60)
Seductive Details Condition	2.11 (2.31)	0.58 (0.70)

**Table 4:** Interview participants.

Condition	Participant ID	Nationality	Academic Year
Control (C)	S1	Korean	Senior
Seductive Details (SD)	S2	French	Senior
	S3	Swedish	Senior
Elaborative Details (ED)	S4	German	Junior
	S5	Uzbek	Sophomore
	S6	Korean	Senior

## Data Availability

The raw data supporting the conclusions of this article will be made available by the authors on request.

## Supplementary Materials

The following supporting information can be downloaded at: https://www.mdpi.com/article/10.3390/jintelligence14050074/s1, Elaborative and Seductive Detail Comparisons.

## Abbreviations

The following abbreviations are used in this manuscript:

CTML	Cognitive theory of multimedia learning
CLT	Cognitive load theory
SI	Situational interest
