# Supplementary material for: Cognitive Load and Working Memory in Multimedia Video Podcasts: Effects of Elaborative and Seductive Details
Source: J Intell. 2026 May 1;14(5):74. doi: 10.3390/jintelligence14050074 (PMC13207565; doi:10.3390/jintelligence14050074)
Supplement: Supplementary file 1 [file jintelligence-14-00074-s001.zip › jintelligence-4146426-supplementary.pdf]

Script Number	Elaborative Details	Seductive Details
2. As this air warms up near the surface, it begins to rise rapidly.	This is similar to a hot-air balloon lifting off the ground when the air inside it heats up.	Speaking of this process, did you know that a single strong updraft in a thunderstorm can propel air upward at nearly 100 miles per hour—the same speed as a professional baseball pitch?
3. And as the air in this updraft cools, it forces the water vapor to condense into water droplets that forms a cloud.	These updrafts are like nature's elevator, lifting the moisture up above our heads.	Did you know that without plentiful dust, salt, small pieces of ash for water vapor to condense around, some clouds could not form at all?
4. The cloud's top extends above the freezing level, so the upper portion of the cloud is composed of tiny ice crystals.	Inside these massive clouds, you have a watery world below and an icy kingdom above.	Sometimes, clouds can reach heights of 12,000 meters or more, and the temperatures in the top can plummet to -40°C.
6. Raindrops and ice crystals fall through the cloud, dragging some of the air in the cloud creating downdrafts	Again, it is like nature's elevator in reverse, carrying the cloud down as raindrops and ice crystals pull the air along for the ride.	Did you know that some thunderstorm updrafts are so powerful they can keep hailstones suspended in the air—giving them time to grow larger before falling to earth?
8. The rising and falling air currents cause electrical charges to build.	This is just like when you rub a balloon on your hair, and it sticks from the static electricity.	Fascinatingly, the energy released by a single lightning bolt can power the average home for several days.
10. Negatively charged particles fall to the bottom of	This splits the cloud into zones of opposite charge,	Sometimes, electric fields are created within the cloud that are strong enough to exceed

the cloud. Lighter, positively charged particles rise to the top.	turning it into a giant floating battery.	100,000 volts per meter—more than 1,000 times stronger than the electric field typically experienced at Earth's surface!
11. A stepped leader of negative charges moves downward in a series of steps-	It is similar to an invisible path of negative charges that zigzag downward from the cloud in a series of "steps", though we cannot see it with the naked eye.	These steps race toward the ground at speeds up to a hundred thousand meters per second, the stepped leader branches out like the roots of a tree.
